# Leveraging ensemble convolutional neural networks and metaheuristic strategies for advanced kidney disease screening and classification

**DOI:** 10.1038/s41598-025-93950-1

**Published:** 2025-04-11

**Authors:** Abeer saber, Esraa Hassan, Samar Elbedwehy, Wael A. Awad, Tamer Z. Emara

**Affiliations:** 1 Information Technology Department, Faculty of Computers and Artificial Intelligence, Damietta University, Damietta, Egypt; 2 Faculty of Artificial Intelligence, Kafrelsheikh University, Kafrelsheikh, 33511 Egypt; 3 Department of Data Science, Faculty of Artificial Intelligence, Kafrelsheikh University, Kafrelsheikh, 33511 Egypt; 4 Computer Science Department, Faculty of Computers and Artificial Intelligence, Damietta University, Damietta, Egypt

**Keywords:** Computed tomography, Deep learning, Optimization, Transfer learning, Convolutional neural network, Computational biology and bioinformatics, Kidney diseases

## Abstract

To address the public health issue of renal failure and the global shortage of nephrologists, an AI-based system has been developed to automatically identify kidney diseases. Recent advancements in machine learning, deep learning (DL), and artificial intelligence (AI) have unlocked new possibilities in healthcare. By harnessing these technologies, we can analyze data to gain insights into symptoms and patterns, ultimately facilitating remote patient care. To create an AI-based diagnosis system for kidney disease, this paper focused on the three major categories of kidney diseases: stones, cysts, and tumors, which were collected and annotated on 12,446 computed tomography (CT) whole abdomen and urogram images. To effectively aid in the automatic identification and diagnosis of kidney diseases, a novel DL model built on the transfer-learning (TL) technology is implemented in this work. DL models are designed to focus on problems, whereas TL uses the knowledge acquired while resolving one issue to another pertinent issue. The proposed model combines multiple DL models to improve overall performance by leveraging the strengths of different architectures, ensembles can enhance accuracy, robustness, and generalization. It enhances the features extracted from MobileNet-V2, ResNet50, and EfficientNet-B0 networks using metaheuristic algorithms and bidirectional long-short-term memory (Bi-LSTM) from the CT image. MobileNetV2, ResNet50, and EfficientNet-B0 hyperparameters have been optimized using a modified grey wolf optimization (GWO) approach for better performance. The suggested model’s performance has been measured using five assessment metrics: accuracy, sensitivity, specificity, precision, and area under the ROC curve (AUC) and achieved 99.85% accuracy, 99.8% sensitivity, 99.3% specificity, 98.1% precision, and 1.0 AUC.

## Introduction

Chronic Kidney Disease (CKD) is a global challenge with over one billion affected by the disease^1,2^. It is expected to be the 16th cause of death in 2040^3^. The common kidney diseases are renal cell carcinoma, cysts, and nephrolithiasis. Simple renal cysts are minor sac- or bladder-like structures lined with epithelium that contain clear fluid and are not part of the kidney and, in many terms, cause harm to the body. Kidney stones, which are prevalent in approximately 12% of the global population, form when the crystals in the kidneys create discomfort and other health issues^4^. Renal cell carcinoma, as a primary malignant tumor, is among the leading cancers and plays a significant role in the development of kidney disease. Both kidney anomalies are necessary to detect early to avoid kidney failure. Nevertheless, the diagnosis of kidney disease is a rather challenging and lengthy process; sometimes it not only requires the urine test and blood test but also computed tomography (CT) scans, ultrasonography (UT), and magnetic resonance imaging (MRI)^5–7^. Even though sophisticated imaging techniques are available in the healthcare system, the delay in diagnosis and human error are potential problems^8,9^. The shortage is particularly acute in the developing world, where access to healthcare is a significant concern, and the current ratios of nephrologists to the population as well as radiologists to the population are significantly wanting. artificial intelligence (AI) and deep learning (DL) help the fight against illnesses through medical aid, notification, screening the population, and helping with infection control^8^. As a scientific technology, it has the potential of enhancing the way renal diseases are managed, anticipated, and communicated. Rather, DL-based biomedical image analysis is emerging as a highly required field at the interface of AI and healthcare^10,11^. Remaining to the inherent nature of biology, data modalities, dataset size, and tasks in biomedical image analysis could hugely differ^12–20^. Different sensors and imaging approaches can be applied to many of the biomedical imaging systems to fit the requirements of applications^21,22^. In this paper, we are interested in the high-resolution CT kidney sensor devices applied in imaging kidneys, noting abnormalities would inform diagnosis of kidney diseases, in addition to other diagnostic algorithms and predicting instruments. The proposed model integrates several advanced techniques to enhance the accuracy of kidney disease classification from CT images. It combines feature extraction from well-established convolutional neural network (CNN) architectures, such as MobileNet-V2, ResNet50, and EfficientNet-B0, with the power of metaheuristic algorithms and Bidirectional Long Short-Term Memory (Bi-LSTM) networks. The following details outline the contributions of each component to the model. By integrating two advanced architectures and optimization methods, the developed model can learn more features from the CT images that are relevant to the diseases, hence improving the classification of the diseases on kidney computing. My proposed models of MobileNet-V2, ResNet50, EfficientNet-B0, and Bi-LSTM with GWO enhance the feature extraction strategy as well as fine-tune the models for better diagnosing kidney-related conditions.

Researchers have applied image-based techniques, such as CT, to diagnose kidney conditions like tumors, cysts, and stones. By incorporating DL techniques, they aim to improve the specificity and sensitivity of diagnoses, reduce early onset of subjective fatigue, and provide a practical framework for identifying patterns in CT scans. Following the modern innovation, especially with regards to AI coupled with DL in analyzing medical images, we propose to determine the use of the AI model in diagnosing radiological alterations in the kidney scan. The concept of transfer-learning (TL) from other related natural image datasets, like the ImageNet, is employed for fine-tuning the DL models to identify kidney disease where databases of medical images are scarce. In addition, simulations from metaheuristics as models derived from natural phenomena of optimization are also incorporated in the DL system to enhance results.

(I) Enhancing the pre-trained network by improving the optimizer and classifier. The Grey Wolf Optimizer (GWO) can be used to find the optimal hyperparameters for the model.
(II) Combining CNNs and Bi-LSTMs into a hybrid architecture allows for leveraging the advantages of both models, with Bi-LSTM capturing long-term dependencies in the data, which is beneficial for kidney classification tasks.
(III) Improving kidney classification performance by using ensemble methods, which combine the predictions of multiple models.

This paper is organized as follows: Section “Related works” summarizes relevant research, Section “The proposed model” outlines a proposed kidney screening and classification model using TL techniques, Section “Results” compares experimental results to real-world data, and Section “Conclusion” concludes the study.

## Related works

As processing power has expanded in recent years, we have observed great growth in development across many industries. DL-based technologies, CNNs, which have been effective in biomedical image processing, are among those that have flourished and advanced. For several medical image analysis applications, such as medical picture detection, abnormality classification, and medical image retrieval, it has come to the top of the list^19^. The topic of biomedical image segmentation using DL has seen the emergence of many exciting algorithms.

Using the kidney stones, cysts, and tumors dataset, Rui et al.^23^ trained a DL model to improve the kidney disease classification. The presented model achieved an overall accuracy of 98%. Mehedi et al.^24^ proposed a model based on transferring the learned parameters from pre-trained models (MobileNetV2, VGG16, and InceptionV3) and achieved 95.29%, 99.48%, and 97.38% accuracy for the three models, respectively. While Md Nazmul^25^ et al. proposed three other models based on Swin transformers, EANet, and CCT. The swin transformer accuracy was 99.30%. Using the same dataset, Abdalbasit and Dana^26^ presented a DL model based on transferring the learned parameters from the Densenet-201 model and a random forest algorithm for classification. The presented method achieved a 99.4% accuracy rate.

Also, Sudharson et al.^10^ introduced a TL model using the same dataset for kidney disease classification from patient ultrasound images. Instead of using separate models, predictions from numerous CNNs result in the best classification performance. The model classified the kidney ultrasound images into four categories with a classification accuracy of 95.58%.

Asif et al.^27^ presented a TL model to effectively identify severe kidney illnesses from CT images, a deep TL methodology based on the VGG19 model, and a simple Inception module was developed. To prevent vanishing gradient and overfitting problems, as well as to enhance performance, the proposed model modified the architecture of the VGG19 by initializing a nave Inception module and other layers. The outcomes demonstrated that when combined with a fine-tuning approach, the suggested model achieved a high accuracy of 99.25%. Kadir et al.^11^ used coronal CT scans with DL to detect kidney stones and achieved an overall accuracy of 96.82%. Fuladi et al.^28^ proposed a DL methodology to automate kidney condition recognition, aiding radiologists in diagnostic tasks. Their methodology entails preprocessing CT images, extracting relevant features, and employing a CNN model for classification. Notably, their model surpasses earlier methodologies, achieving an impressive 99.57% accuracy, 99.34% F1-score, 99.56% recall, and 99.58% precision. This promising performance suggests a potential for further advancements in the field.

From a U.S. database, Tsai et al.^29^ gathered 330 normal and 1269 aberrant pediatric renal pictures. The final connecting layer of ResNet50 was fine-tuned after completing the pre-processing tasks, and an accuracy of 92.9% was obtained.

## The proposed model

**Fig. 1:**
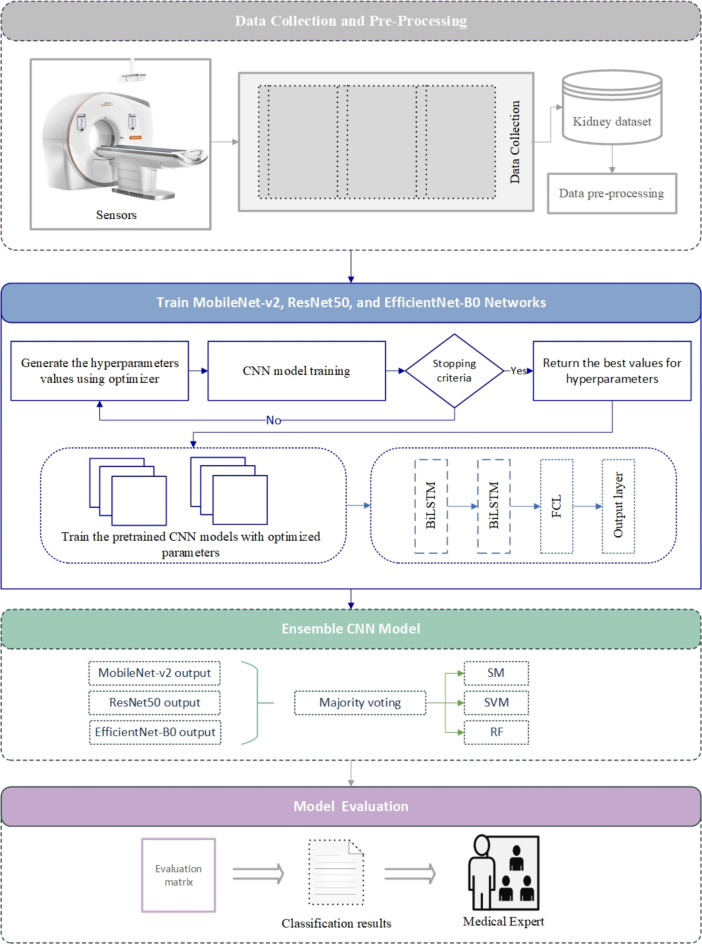
The proposed model.

An ensemble-based DL model for kidney identification is given in this work. The entire architecture is shown in Fig. 1. Participants’ radiogram images were obtained using medical sensors and X-ray machines, and the data set was later utilized to diagnose kidney disorders using the proposed model. The presented model for kidney disease detection and classification contains four main components.

**Fig. 2:**
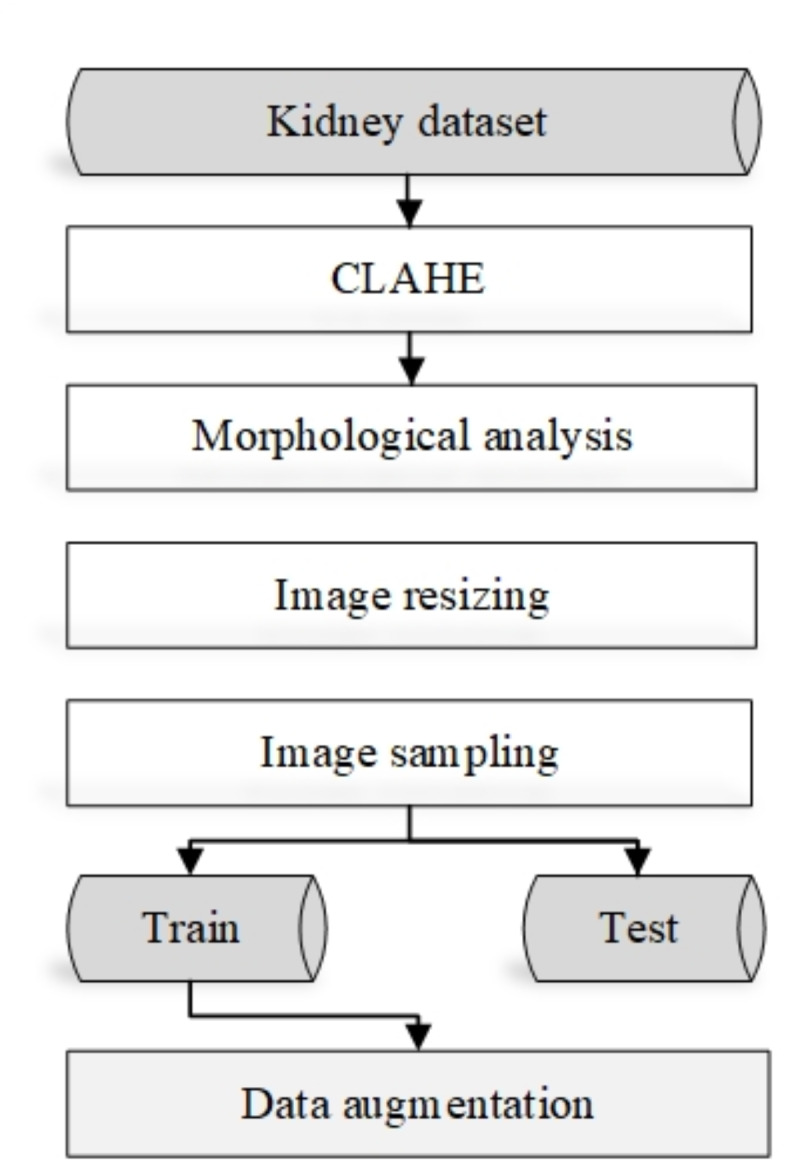
The data pre-processing steps.

### Data pre-processing steps

The data preprocessing steps contain five phases as indicated in Fig. 2.

#### Constructed limited histogram equalization (CLAHE)

Instead of processing a complete image, CLAHE only works on discrete or tiled portions. The surrounding tiles blended using bilinear interpolation to eliminate the erroneous boundaries. Image contrast can be improved using this algorithm^30^.

#### Morphological analysis

To ensure that the results are unaffected, it is crucial to exclude non-kidney regions using the morphological analysis. A structural element is added by morphological processes to an input image to generate an identical-sized output image. Each output pixel’s value is identified by comparing it to its neighbors in the input image when executing a morphological operation^31^.

#### Image resizing

All images are scaled down to 224 × 224 pixels and adjusted to have three color channels, aligning with the input dimensions of the pre-trained CNNs.

#### Image sampling

The proportions are 80% for training and 20% for testing.

#### Data augmentation

For increasing the input data, the is used. The kidney CT images are rotated at 45°, 90°, 180°, and 270°. Next, each rotated picture is flipped vertically. Eight images will result from a single input image in this way.

### Transferring the learning parameters

This paper uses the ensemble pre-trained MobileNet-V2, ResNet50, and EfficientNet-B0 networks to improve the kidney disease classification task. The ImageNet dataset is used to train these networks. The network layers’ filters are applied to realize input features. As a result, trivial shapes and small parts can be recognized. The produced output can be used to determine the class to which the input image belonged.

To classify kidney diseases, we employed an ensemble of pre-trained CNNs. This powerful technique combines multiple models to enhance overall performance. Our ensemble comprised three state-of-the-art CNN architectures: ResNet50, EfficientNet-B0, and MobileNet-V2. To optimize the ensemble’s performance, we fine-tuned the models using three popular optimization algorithms: Stochastic Gradient Descent with Momentum (SGDM), Adaptive Moment Estimation (Adam), and GWO. By leveraging the strengths of these diverse models and optimization techniques, we aimed to achieve superior accuracy, robustness, and generalization on a new dataset of kidney disease images.

In the proposed network, rather than sending the sequence of deep features directly to the fully connected layer, it sends it first to the Bi-LSTM layer before applying SM, SVM, and RF algorithms for classification. The pre-trained networks efficiently extract and recognize the image’s local and global structures in the pixel series, whereas the Bi-LSTM network discovers long-short-term dependencies that are vital for accurate interpretation.

Long-term dependencies in CT images are vital for accurate interpretation and analysis, as these images consist of slices representing cross-sections of the body, where relationships between pixels across slices can reveal important anatomical features, such as tumors that span multiple slices. Additionally, in monitoring disease progression over time, it’s crucial to capture dependencies across different imaging sessions. The complexity of anatomical structures often necessitates considering surrounding slices to enhance recognition and classification tasks, as isolated slices may not provide a complete picture.

Bi-LSTMs enhance model performance in processing CT images by utilizing bidirectional processing, which allows them to capture dependencies from both past and future slices, leading to a more comprehensive understanding of the image context. This bidirectionality results in richer feature representations, improving the accuracy of tasks like segmentation and classification. Additionally, Bi-LSTMs address the vanishing gradient problem, enabling the retention of information over longer sequences, which is crucial for the complex nature of CT images. Finally, they effectively model the temporal progression of a series of scans, facilitating the learning of patterns indicative of disease progression or treatment responses. The pseudocode is illustrated in Algorithm 1.

**Algorithm 1:**
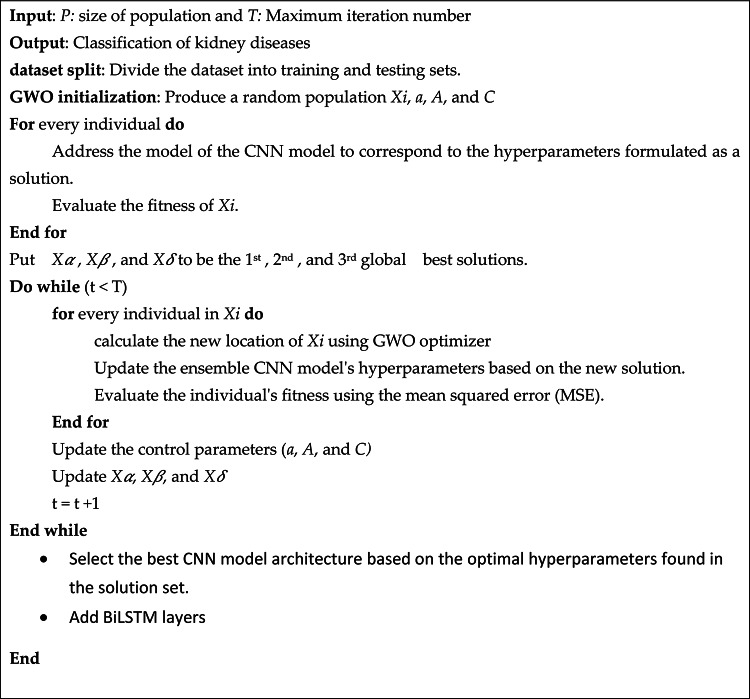
Pseudo code of the optimized CNN model using GWO and LSTM layers

### The deep optimized CNN

#### Ensemble CNN learning

Ensemble learning is a technique that involves training multiple machine learning models and combining their predictions. It’s like having a “committee” of experts making decisions together. By combining the strengths of individual models, ensemble learning can often achieve higher overall accuracy than any single model would on its own^32–35^.

The Ensemble model merged three distinct neural network architectures: ResNet50 + EfficientNet-B0 + MobileNet-V2. These subnetworks were integrated into a larger neural network with multiple heads, enabling the model to learn more effectively from the combined predictions of each individual submodel. This approach treats the stacked submodels as a single, unified model.

To combine the outputs of each model, a simple concatenation merge neural network was employed. The probabilities predicted by each model were concatenated into a single vector, which was then processed through a hidden layer using the ReLU activation function. Finally, an output layer with classifier was used to separate the classes and provide probabilistic predictions. The pseudocode for the ensemble model is illustrated in Algorithm 2.

**Algorithm 2:**
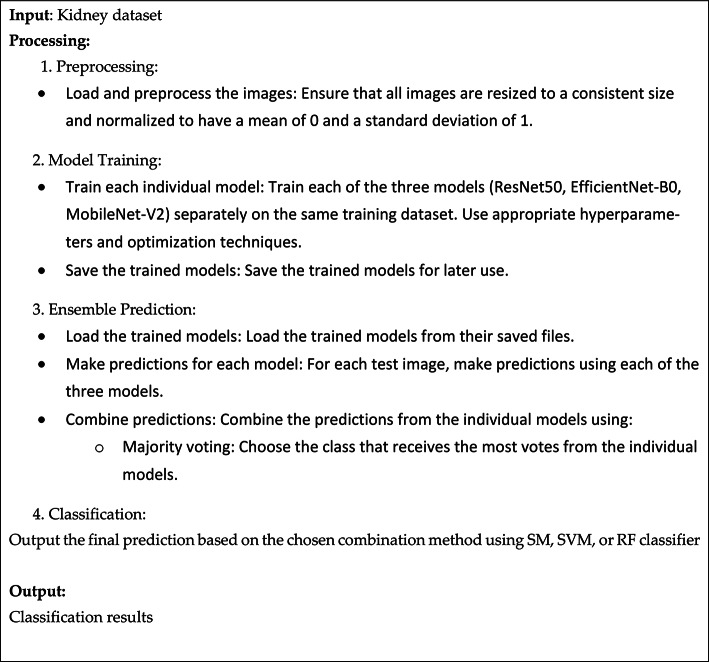
Pseudo code of the Ensemble model.

The basis of MobileNetV2 is an inverted residual structure with residual connections connecting the bottleneck levels. The 32-filter initial fully convolution layer is followed by 19 more bottleneck layers in the MobileNetV2 design^36^.

EfficientNet is a family of CNNs designed to achieve high performance while maintaining efficiency. EfficientNetB0 is a basic model, similar in size to a mobile network. The other models in the family are created by systematically increasing the model size using an effective scaling method to maximize accuracy gains^37^.

ResNet50 is a deep neural network architecture with 50 weighted layers. It was designed to overcome the limitations of shallower networks, which often struggle to achieve high accuracy in classification tasks. The ResNet architecture is illustrated in^3^.

#### The metaheuristic algorithms

A. Grey Wolf Optimizer.

The mathematical formulas of GWO are discussed and presented in this subsection. GWO mimics the encircling, tracking, social hierarchy, and preys’ attacking^38^.

i. Social hierarchy;

To create the GWO algorithm, the social hierarchy of wolves is mathematically modelled. The alpha ($\:\alpha\:$) is the most robust solution, followed by beta ($\:\beta\:$) and delta ($\:\delta\:$) as the second and third-best solutions, respectively. The remaining solutions are considered omega ($\:\omega\:$). The alpha, beta, and delta wolves guide the optimization process, while the omega wolves follow their lead. This forms the basis of the GWO algorithm’s approach to optimization.

ii. Encircling prey;

The encircling behaviour of grey wolves during hunting can be mathematically modeled using the following equations:

1 $$\overrightarrow{D}=\overrightarrow{|C}\:.\:{\:X}_{P}\left(t\right)-\overrightarrow{X}\left(t\right)|$$

2 $$\overrightarrow{X}\left(t+1\right)={\overrightarrow{X}}_{p}\left(t\right)-\overrightarrow{A}.\overrightarrow{D}$$

where $t$ refers to the current number of iterations, $\:{\overrightarrow{X}}_{p}$ refers to the prey location vector, $\:\overrightarrow{X}$ denotes the location of an individual wolf, and $\:\overrightarrow{A}$ and $\:\overrightarrow{C}$ can be computed from the two following equations:

3 $$\:\overrightarrow{A}=2\overrightarrow{a}.{\overrightarrow{r}}_{1}-\overrightarrow{a}$$

4 $$\:\overrightarrow{C}=2.{\overrightarrow{r}}_{2}$$

Where the vector a is linearly reduced from 2 to 0 as the number of iterations increases. $\:{\overrightarrow{r}}_{1}$ and $\:{\overrightarrow{r}}_{2}$are random vectors ranging from 0 to 1.

iii. Hunting;

Grey wolves possess the instinct to detect the prey’s location and move around them to encircle. However, in an abstract search space, it is impossible to know the exact location of the optimum (prey). To mathematically model the hunting behavior of grey wolves, the GWO algorithm considers the alpha as the best solution, with beta and delta also having some knowledge of the position of potential prey. Therefore, the best three answers are saved, and the rest of the search agents (omegas) are required to update their positions based on the position of the best search agents. The following equations are illustrated to simulate this behavior. The subsequent equations are used to calculate the positions of the leaders.

5 $$\:{\overrightarrow{D}}_{\alpha\:}=|{C}_{1}.\:{\overrightarrow{X}}_{\alpha\:}-\overrightarrow{A}|$$

6 $$\:{\overrightarrow{D}}_{\beta\:}=|{C}_{2}.\:{\overrightarrow{X}}_{\beta\:}-\overrightarrow{A}|$$

7 $$\:{\overrightarrow{D}}_{\delta\:}=|{C}_{3}.\:{\overrightarrow{X}}_{\delta\:}-\overrightarrow{A}|$$

8 $$\:{\overrightarrow{X}}_{1}=|\:{\overrightarrow{X}}_{\alpha\:}-{\stackrel{-}{A}}_{1}.{\overrightarrow{D}}_{\alpha\:}|$$

9 $$\:{\overrightarrow{X}}_{2}=|\:{\overrightarrow{X}}_{\beta\:}-{\stackrel{-}{A}}_{2}.{\overrightarrow{D}}_{\beta\:}|$$

10 $$\:{\overrightarrow{X}}_{3}=|\:{\overrightarrow{X}}_{\delta\:}-{\stackrel{-}{A}}_{3}.{\overrightarrow{D}}_{\beta\:}|$$

11 $$\:\overrightarrow{X}\left(t+1\right)=\frac{{\overrightarrow{X}}_{1}+{\overrightarrow{X}}_{2}+{\overrightarrow{X}}_{3}}{3}$$

12 $$\:\overrightarrow{X}\left(t+1\right)={w}_{1}{\overrightarrow{X}}_{1}+{w}_{2}{\overrightarrow{X}}_{2}+{w}_{3}{\overrightarrow{X}}_{3}\:\text{w}\text{h}\text{e}\text{r}\text{e}\:\sum\:_{i=1}^{3}{w}_{i}$$

iv. Exploration;

Wolves are instinctive to detect the prey’s location and move around them to encircle. However, in an abstract search space, knowing the exact optimum (prey) location is impossible. To create a mathematical model of how grey wolves hunt, the GWO algorithm considers the alpha as the best solution, with beta and delta also having some knowledge about the potential location of prey. To mathematically model the hunting behavior of grey wolves, the GWO algorithm considers the alpha as the best solution, with beta and delta also having some knowledge about the potential location of prey. Consequently, the top three solutions are saved, and the rest of the search agents (omegas) are required to modify their positions based on the best search agents’ positions.

v. Exploitation;

The grey wolves in GWO mostly follow the alpha, beta, and delta to search for prey but also diverge from them to explore the search space. To model this divergence behavior, the coefficient vector $\:\overrightarrow{A}$ is used with random values > 1 or < -1, which encourages search agents to explore globally. Using $\:\overrightarrow{C}$ with random values in [0, 2] is another component that promotes exploration and avoids local optima. This vector provides random weights for prey, which can either emphasize or deemphasize their effect in defining the distance. Unlike $\:\overrightarrow{A}$, the values of $\:\overrightarrow{C}$ are not linearly decreased, and random values are used throughout the optimization process to emphasize exploration in both initial and final iterations. This component is beneficial when the optimization process stagnates at local optima.

GWO can significantly enhance the performance of CNNs by optimizing their weight parameters through a novel search strategy inspired by the social hierarchy and hunting mechanisms of grey wolves. In this approach, GWO employs a population-based technique that balances exploration and exploitation, allowing it to effectively navigate the complex weight space of CNNs. By simulating the leadership and cooperation among wolves, GWO can identify optimal weight configurations that improve feature extraction and classification accuracy. As a result, CNNs optimized with GWO often achieve higher performance metrics, such as increased accuracy and reduced error rates, making them more robust in tasks like image classification and object detection.

B. SGDM optimizer.

SGDM is a powerful optimizer that can significantly improve the training process of neural networks. By introducing momentum, it helps to accelerate convergence and reduce oscillations, leading to better performance^39^.

The Advantages of SGDM are:

i. Faster convergence: The momentum term helps the optimizer avoid getting stuck in local minima or saddle points.
ii. Reduced oscillations: The momentum term smooths out the updates, preventing the optimizer from bouncing back and forth.
iii. Improved performance: SGDM often leads to better results compared to vanilla SGD.

C. Adam optimizer.

Adam is a popular optimization algorithm that combines the best aspects of two other algorithms: Adaptive Gradient Algorithm (AdaGrad) and Root Mean Square Propagation (RMSprop). It’s widely used in DL due to its effectiveness and efficiency.

The Advantages of Adam are:

i. Adaptive learning rates: Adam adapts the learning rate for each parameter, allowing for faster convergence.
ii. Efficient optimization: Adam is often more efficient than other optimizers, especially for large models and datasets.
iii. Robustness: Adam is relatively robust to different learning rates and hyperparameters.

## Results

### The dataset description

The dataset was acquired from several hospitals in Dhaka, Bangladesh, where individuals had already been classified as having kidney stones, cysts, tumors, or normal results. The region of interest for each radiological discovery was then prepared in a batch of Dicom images, classified, and transformed into a lossless Joint Photographic Experts Group (JPEG) image format^40^.

**Fig. 3:**
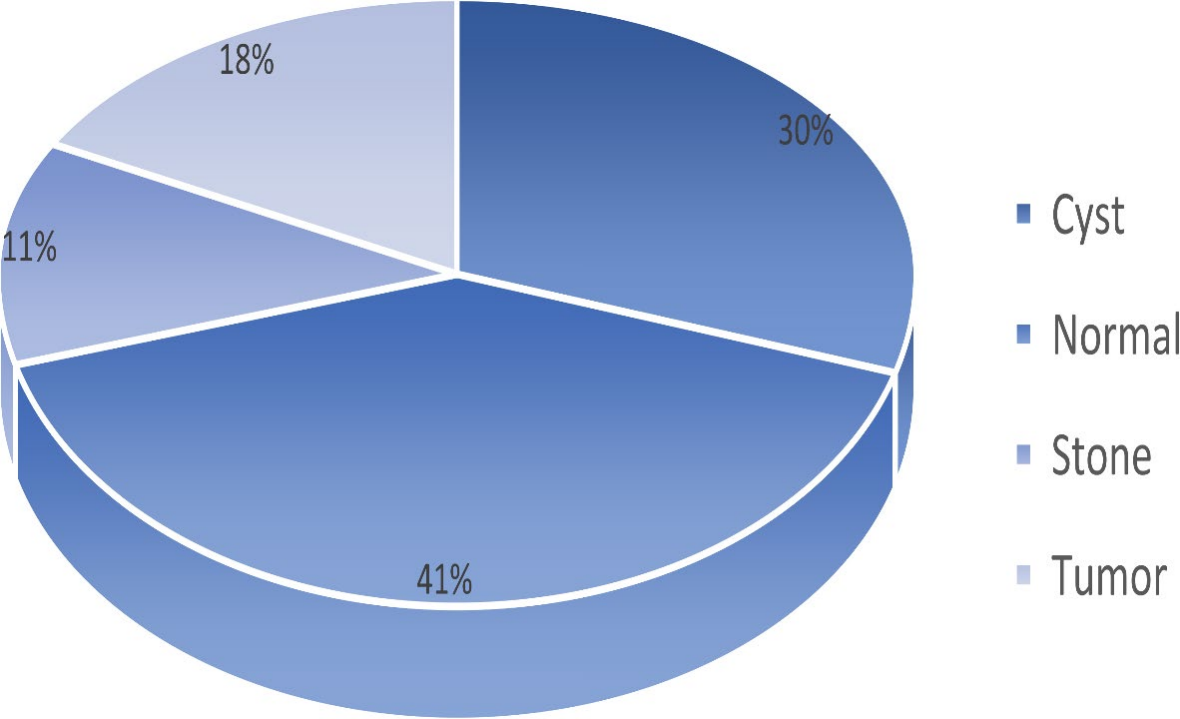
Dataset description.

**Fig. 4:**
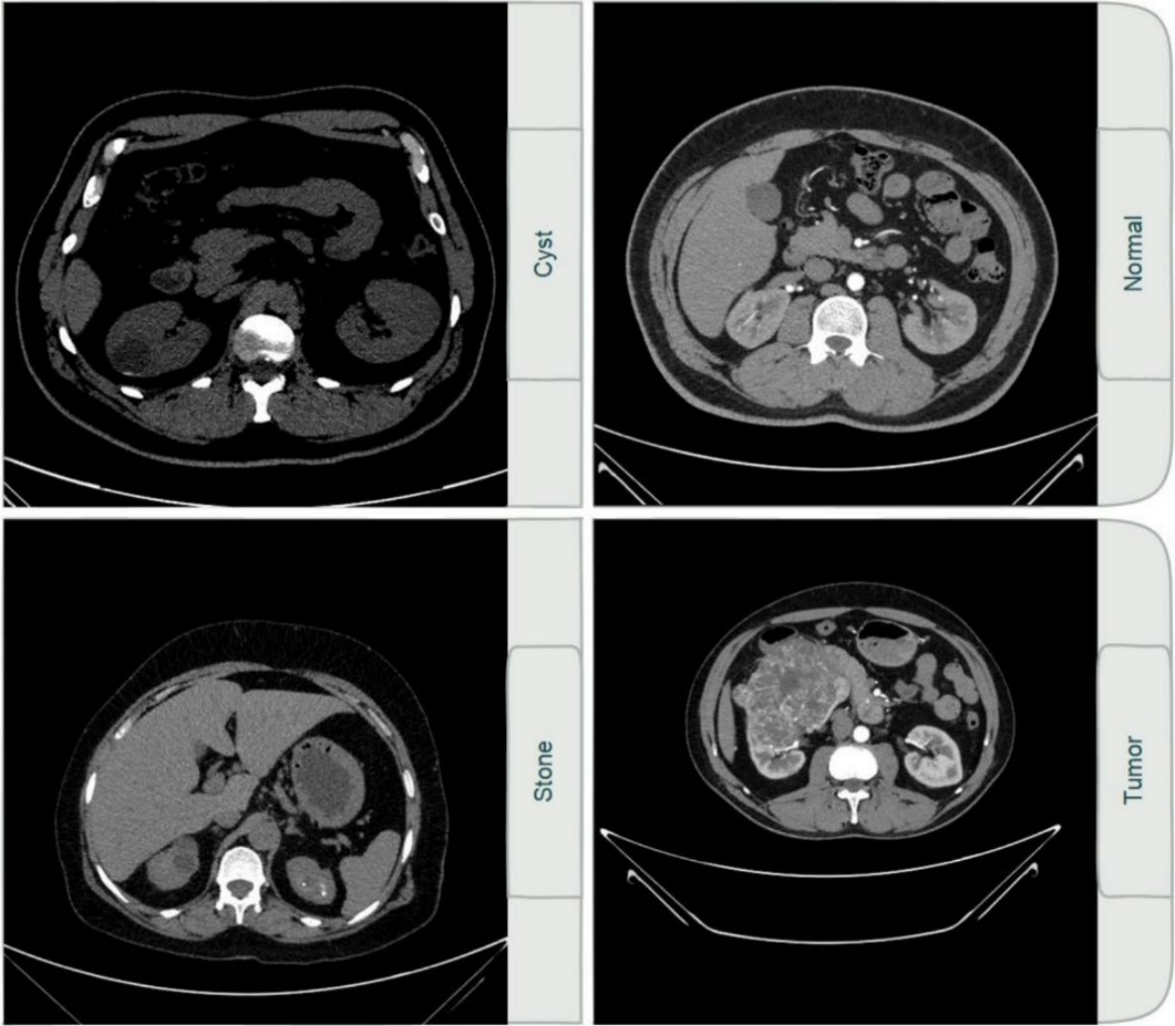
Dataset samples.

The dataset has 12,446 distinct images, of which 3,709 are cysts, 5,077 are normal, 1,377 are stones, and 2,283 are tumors. The dataset description is shown in Fig. 3. The dataset samples are shown in Fig. 4.

### Experimental results

This section outlines several tests conducted to evaluate the performance of the proposed model on the kidney dataset. Specifically, the ensemble CNN model, enhanced with the GSDM, Adam, and GWO optimizers, is assessed and compared using key metrics, including accuracy, precision, sensitivity, specificity, and AUC. The four classes of the dataset were “Normal, Cyst, Tumor, and Stone.” Once divided, the training and testing tasks received 80% and 20% of the total time, respectively. The evaluation indicators for four classes were used to gauge the suggested models’ efficacy.

The benefits of preprocessing were investigated by performing experiments twice, once before and once after preprocessing, as indicated in Tables 1, 2 and 3.

**Fig. 5:**
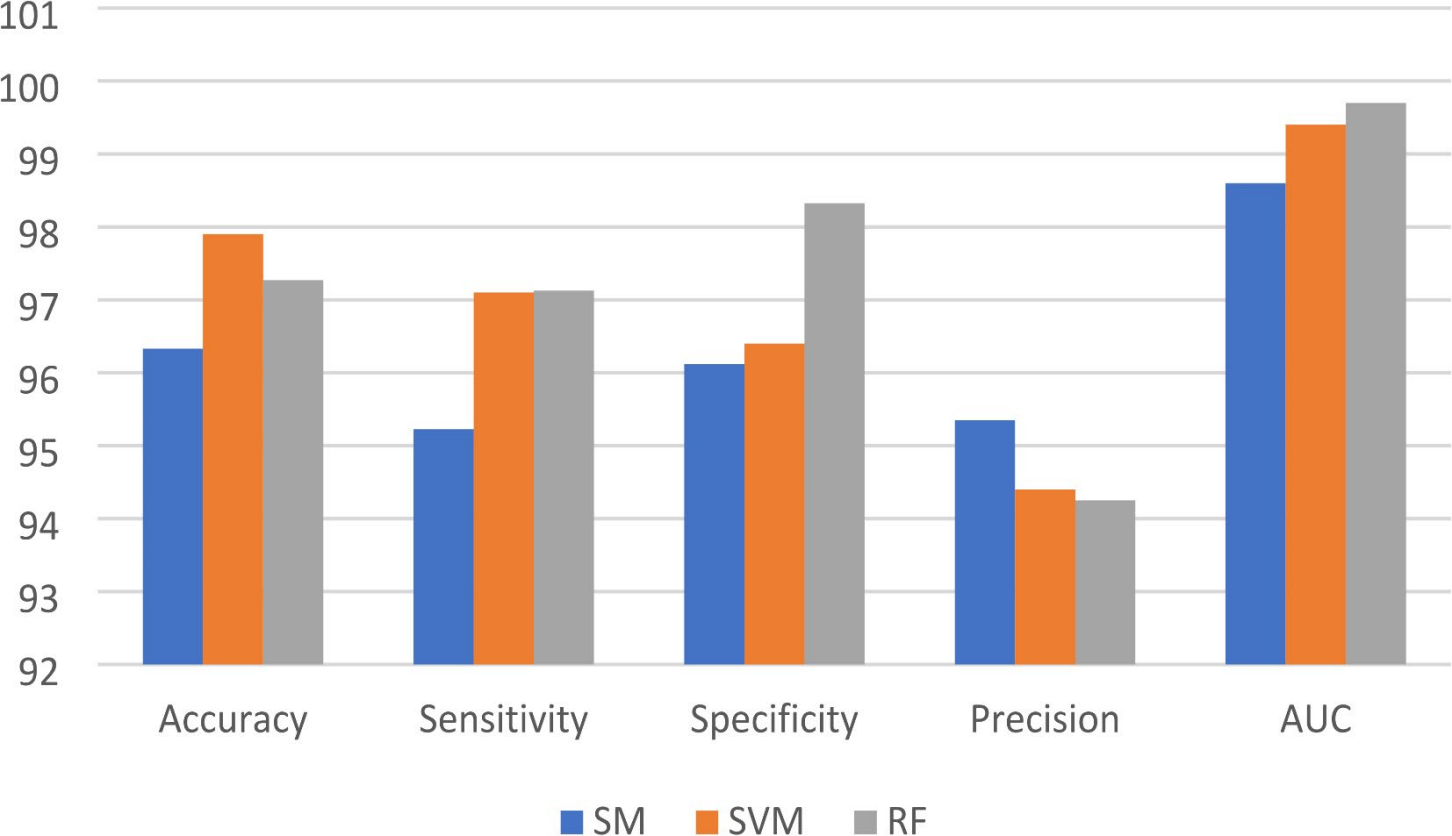
The comparison between optimized ensemble CNN results by SGDM with three classifiers.

**Fig. 6:**
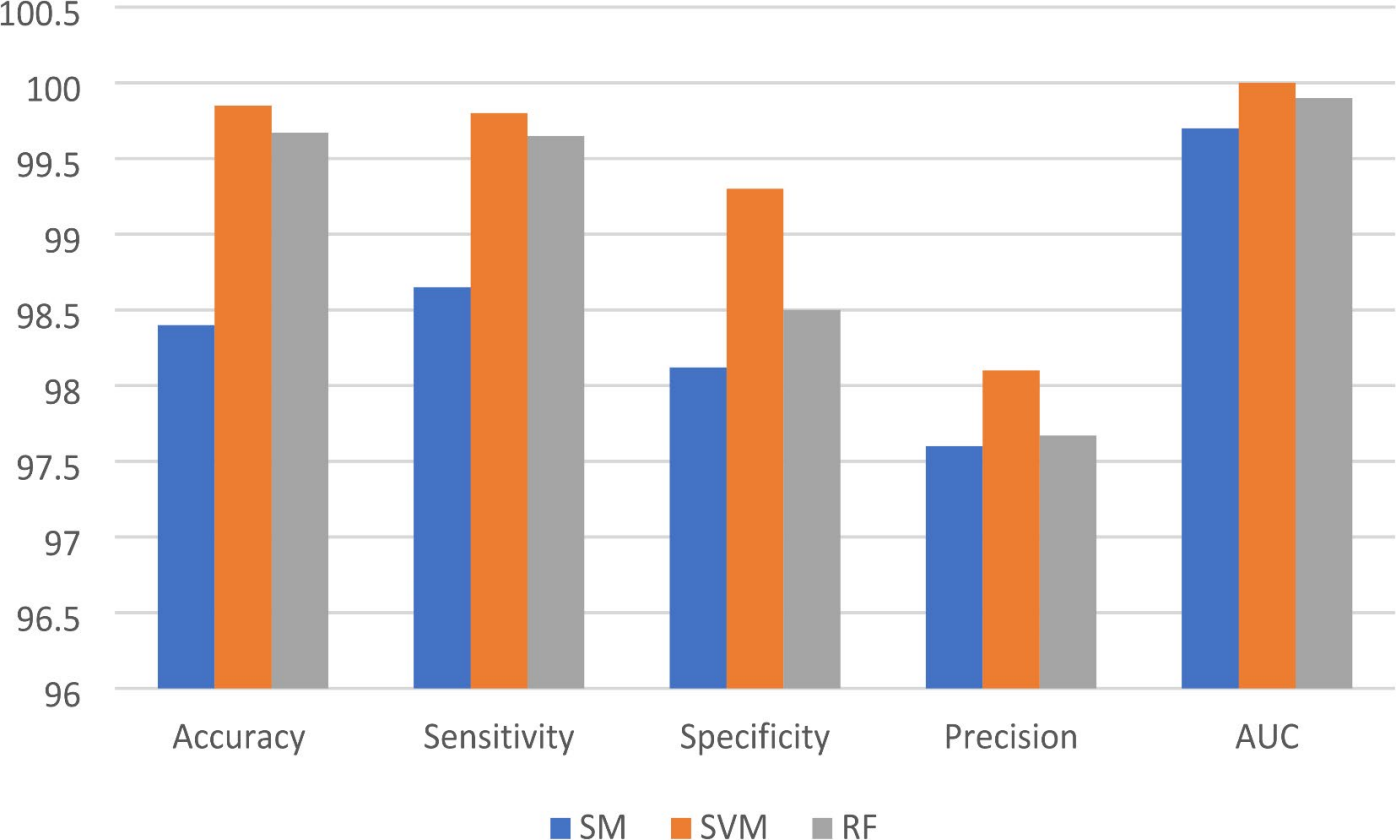
The comparison between optimized ensemble CNN results by Adam with three classifiers.

**Fig. 7:**
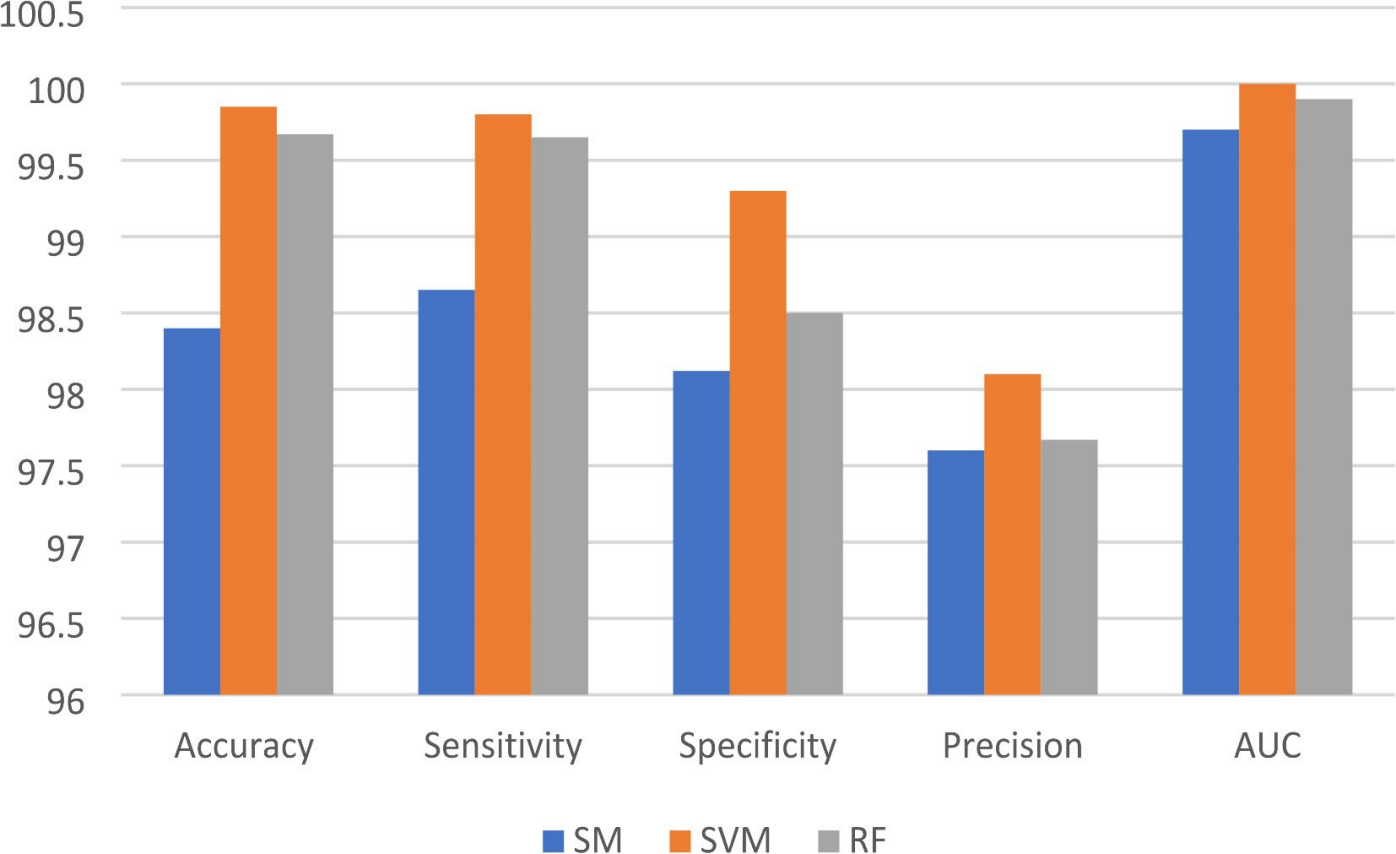
The comparison between optimized ensemble CNN results by GWO with three classifiers.

**Fig. 8:**
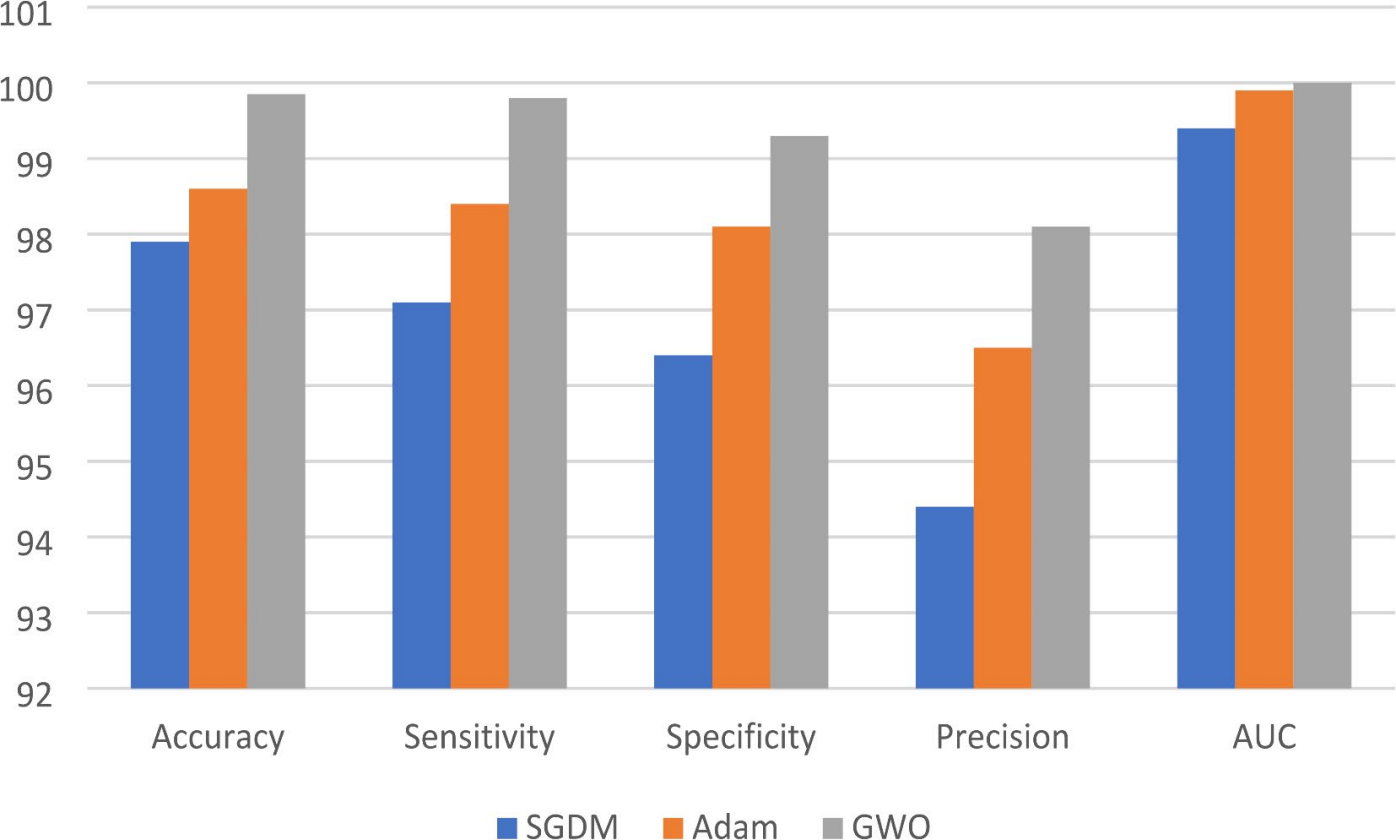
Comparison between the best results achieved by three optimization algorithms.

**Fig. 9:**
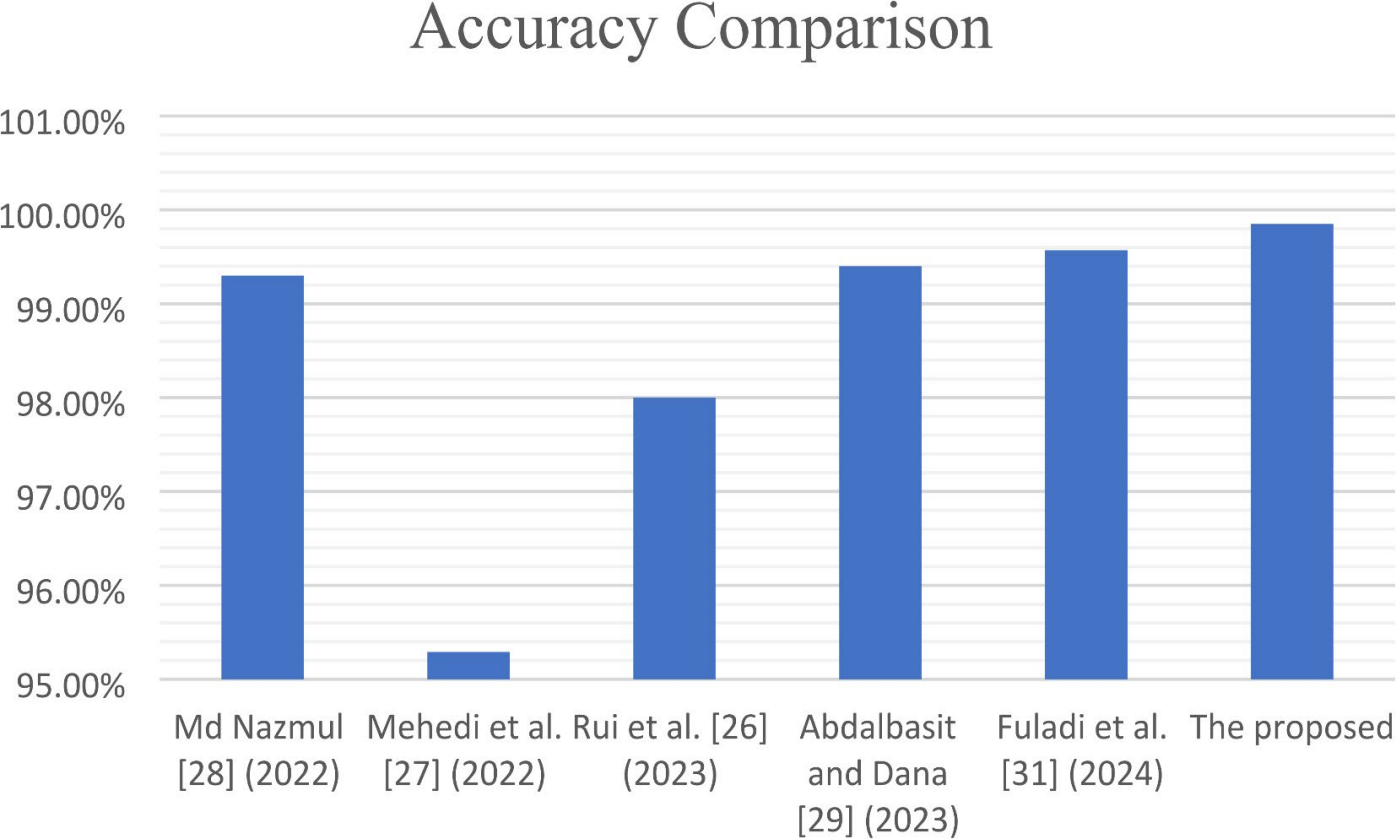
Comparison between related works and the presented model based on the same dataset.

Table 1 Shows the results before and after pre-processing using the SGDM optimizer for every class in the kidney database. The obtained features are extracted and classified using Softmax, MSVM, and RF classifiers. The best results were achieved by SVM with 97.9%, 97.1%, 96.4%, 94.4%, and 0.994 for accuracy, sensitivity, specificity, precision, and AUC, respectively. Table 2 indicated that the accuracy, sensitivity, specificity, precision, and AUC values can be improved by extracting features using the Adam optimizer, achieving 98.6%, 98.4%, 98.1%, 96.5%, and 0.999, respectively. The comparison between SM, SVM, and RF classifiers is shown in Figs. 5 and 6, and 7. The modified GWO algorithm achieved the best classification results than SGDM and Adam optimizers with 99.85%, 99.8%, 99.3%, 98.1, and 1.0 for accuracy, sensitivity, specificity, precision, and AUC, respectively, as illustrated in Table 3. It can be observed that the SVM classifier achieved the best results in almost all values compared to the SM and RF classifiers. Figure 8 illustrates the comparison between the best results achieved by three optimization algorithms. Figure 9 compares related works with the presented model based on the same dataset.

**Table 1:** The results were applied using the ensemble CNN and SGDM per class.

CNN	Class	Performance of the classifier				
		Accuracy (%)	Sensitivity (%)	Specificity (%)	Precision (%)	AUC
Before pre-processing	Cyst	63.8	65.1	56.1	56.9	0.44
	Normal	59.82	63.7	62.6	62.1	0.45
	Stone	61.63	60.1	58	60.3	0.44
	Tumor	63.76	64.7	66.7	61.3	0.45
	Average	62.25	63.4	60.85	60.15	0.445
After pre-processing (SM)	Cyst	96.82	94.2	95.6	94.1	0.99
	Normal	97.1	95.7	97.4	95.2	0.987
	Stone	95.3	96.1	96.2	95.9	0.981
	Tumor	96.1	94.9	95.3	96.2	0.988
	Average	96.33	95.23	96.12	95.35	0.986
After pre-processing (SVM)	Cyst	98.36	97.2	97.1	95.8	0.994
	Normal	97.9	96.9	95.9	93.3	0.998
	Stone	98.45	96.5	96.5	94.4	0.997
	Tumor	97.1	97.8	96.2	94.1	0.988
	Average	97.9	97.1	96.4	94.4	0.994
After pre-processing (RF)	Cyst	98.1	98.1	98.5	94.1	0.997
	Normal	97.3	95.8	99.2	93.2	0.996
	Stone	96.1	97.7	98.7	95.1	0.997
	Tumor	97.6	96.9	96.9	94.6	0.998
	Average	97.27	97.13	98.33	94.25	0.997

**Table 2:** The results were applied using the ensemble CNN and Adam optimizer per class.

CNN	Class	Performance of the classifier				
		Accuracy (%)	Sensitivity	Specificity	Precision	AUC
Before pre-processing	Cyst	63.9	65.6	65.9	58.8	0.45
	Normal	60.2	64.8	64.4	62.5	0.46
	Stone	64.2	62.2	68.2	61.6	0.44
	Tumor	65.1	64.2	64.3	59.7	0.43
	Average	63.35	64.2	65.7	60.65	0.44
After pre-processing (SM)	Cyst	97.1	97.6	98.1	94.9	0.998
	Normal	98.2	95.9	97.9	96.3	0.998
	Stone	97.9	97.1	96.6	95.6	0.995
	Tumor	96.6	96.4	96.9	96.8	0.996
	Average	97.45	96.75	97.38	95.9	0.997
After pre-processing (SVM)	Cyst	98.1	98.1	98.6	97.5	0.999
	Normal	99.1	98.9	98.3	96.6	1
	Stone	98.3	97.9	97.6	96.1	0.999
	Tumor	98.9	98.7	97.9	95.9	0.998
	Average	98.6	98.4	98.1	96.5	0.999
After pre-processing (RF)	Cyst	97.2	98.2	98.4	97.6	0.996
	Normal	99	98.4	97.7	96.5	0.999
	Stone	98.4	98.1	98.1	95.9	0.997
	Tumor	98.3	98.1	97.9	95.5	0.997
	Average	98.2	98.2	98.02	96.37	0.997

**Table 3:** The results were applied using the ensemble CNN and GWO per class.

CNN	Class	Performance of the classifier				
		Accuracy (%)	Sensitivity	Specificity	Precision	AUC
Before pre-processing	Cyst	63.9	65.4	69.1	58.9	0.45
	Normal	62.2	63.8	65.2	63.2	0.46
	Stone	64.9	69.9	68.1	62.4	0.47
	Tumor	65.1	65.1	64.5	59.9	0.46
	Average	64.03	66.05	66.72	61.1	0.46
After pre-processing (SM)	Cyst	98.7	99.5	97.9	97.2	0.997
	Normal	98.8	98.7	98.5	97.9	0.997
	Stone	98.3	98.5	98.4	97.7	0.999
	Tumor	97.9	97.9	97.7	97.6	0.998
	Average	98.4	98.65	98.12	97.6	0.997
After pre-processing (SVM)	Cyst	99.7	100	99.8	97.3	1
	Normal	100	99.7	99.6	98.8	1
	Stone	99.9	100	99.1	97.9	1
	Tumor	99.8	99.5	98.8	98.5	1
	Average	99.85	99.8	99.3	98.1	1
After pre-processing (RF)	Cyst	99.5	100	98.8	97.1	1
	Normal	99.9	99.6	99.1	98.2	0.999
	Stone	99.7	99.8	98.6	97.3	1
	Tumor	99.6	99.2	97.6	98.1	0.998
	Average	99.67	99.65	98.5	97.67	0.999

## Conclusion

In this paper, we propose a robust model for kidney disease classification that has been thoroughly explained and shown to yield significant quantitative improvements. We initially preprocessed conventional CT images by adjusting image contrast. Subsequently, we extracted feature descriptors from this dataset using optimized pre-trained networks. Temporal features were then extracted through a Bi-LSTM, which allowed the model to leverage temporal dependencies and enhance its predictive capabilities. Our proposed method utilizes an ensemble of three CNNs: ResNet50, EfficientNet-B0, and MobileNet-V2. This ensemble approach improves classification accuracy, reliability, and generalization. When combined with an improved GWO algorithm, the ensemble achieved higher accuracy than optimizers like SGDM and Adam. The model delivered near-perfect results, achieving an accuracy of 99.85%, a sensitivity of 99.8%, a specificity of 99.3%, a precision of 98.1%, and an AUC of 1.0.

These outcomes indicate a significant improvement over existing methods applied to the same dataset, demonstrating the efficiency and robustness of our proposed approach. By meticulously tuning model parameters, incorporating advanced DL algorithms, and employing novel evaluation metrics, we have achieved superior results. Additional evaluation criteria, including accuracy, sensitivity, specificity, precision, and AUC, further validate the model’s effectiveness in accurate kidney disease screening and diagnosis. While our proposed method represents a significant advancement in the field, given the limitations of the current dataset, we believe that further research is necessary. Future iterations of this study will explore the integration of external datasets into the validation process to enhance the model’s robustness and generalizability. Additionally, incorporating laboratory test data along with CT images into the model can significantly improve diagnostic accuracy, providing a more comprehensive solution for kidney disease detection in healthcare.

## Data Availability

https://www.kaggle.com/datasets/nazmul0087/ct-kidney-dataset-normal-cyst-tumor-and-stone.
